# Neonatal Mortality and Temperature in Two Northern Swedish Rural Parishes, 1860–1899—The Significance of Ethnicity and Gender

**DOI:** 10.3390/ijerph17041216

**Published:** 2020-02-13

**Authors:** Lena Karlsson, Erling H. Lundevaller, Barbara Schumann

**Affiliations:** 1 Centre for Demographic and Ageing Research (CEDAR), Umeå University, 901 87 Umeå, Sweden; erling.lundevaller@umu.se (E.H.L.);; 2 Department of Sociology, Umeå University, 901 87 Umeå, Sweden; 3 Department of Statistics, Umeå University, 901 87 Umeå, Sweden; 4 Department of Epidemiology and Global Health, Umeå University, 901 87 Umeå, Sweden

**Keywords:** neonatal mortality, temperature, seasonality, indigenous population, gender, Sweden

## Abstract

The aim of this study was to analyze the association between season of birth and daily temperature for neonatal mortality in two Swedish rural parishes between 1860 and 1899. Further, we aimed to study whether the association varied according to ethnicity (indigenous Sami reindeer herders and non-Sami settlers) and gender. The source material for this study comprised digitized parish records from the Demographic Data Base, Umeå University, combined with local weather data provided by the Swedish Meteorological and Hydrological Institute. Using a time event-history approach, we investigated the association between daily temperature (at birth and up to 28 days after birth) and the risk of neonatal death during the coldest months (November through March). The results showed that Sami neonatal mortality was highest during winter and that the Sami neonatal mortality risk decreased with higher temperatures on the day of birth. Male neonatal risk decreased with higher temperatures during the days following birth, while no effect of temperature was observed among female neonates. We conclude that weather vulnerability differed between genders and between the indigenous and non-indigenous populations.

## 1. Introduction

The infant mortality rate has been used as an indicator of the general welfare and population health of a country. In Sweden, nationwide data on infant mortality have been available from the mid-eighteenth century and reveal a downward trend [1]. In nineteenth century Sweden, however, infant mortality was associated with large geographical differences [2,3,4,5]. Mortality rates in northern Sweden in particular, including in the Sápmi area (traditional land of the indigenous Sami), were sometimes as high as 250/1000 live births [5]. One of the factors associated with the high infant mortality rate in the Sápmi area is the harsh climate with long and cold winters [6,7].

In this paper, we studied the association of daily temperature and seasonality with neonatal mortality, covering a sensitive period of an infant’s life. Our previous studies, based on monthly mean temperatures, showed that Sami perinatal and neonatal mortality were influenced by extreme cold in the winter, whereas extreme cold winter temperature had no effect on the non-Sami population [6,8].

In most parts of the world, neonatal and post neonatal mortality are still higher among indigenous populations than non-indigenous populations living in the same area [9,10,11]. In nineteenth century Sweden, the indigenous Sami population experienced a higher neonatal mortality rate compared to the non-Sami population, especially during the first half of the century [12,13]. In historical and contemporary populations, the male disadvantage in neonatal mortality is well recognized [14,15,16,17,18] and is essentially explained by biological factors, such as a higher risk of congenital abnormalities among male new-borns relative to females and a higher incidence of infectious and non-infectious diseases in boys [16,17]. There is a lack of research regarding the influence of temperature on neonatal mortality among boys relative to girls. In this study, we aimed to study whether the association between temperature at birth and daily temperature following birth affects boys and girls differently.

While previous studies of the association between temperature and neonatal mortality among historical populations mainly used month of birth as a proxy for temperature or monthly mean temperatures [6], studies that include daily temperatures are limited [19,20]. In this study, we aimed to unravel the association between local daily temperature conditions and neonatal mortality in two Swedish rural parishes between 1860 and 1899. Further, we aimed to study whether the association varied according to ethnicity and gender.

The study area constitutes a part of Swedish Sápmi, the Sami’s traditional land, inhabited by both Sami and non-Sami populations. This subarctic region represents a climatically and environmentally unique geographical unit, with long, cold winters and short, mild summers. The study included two parishes in northern Sápmi, Jokkmokk and Gällivare. At the beginning of the nineteenth century, the Sami constituted the majority population in the two parishes but had become a minority by the end of the century due to the immigration of settlers from southern Sweden and Finland [21]. In this northern part of Swedish Sápmi, the Sami mainly lived from reindeer herding, hunting, fishing and farming [4]. During the nineteenth century, an increasing part of the Sami population was forced to leave the reindeer herding nomadic lifestyle for a more settled way of living in agriculture [22,23].

### 1.1. Seasonality, Temperature and Infant Mortality

Several studies have investigated the association between season of birth and neonatal mortality among historical populations [24,25,26,27,28]. An important factor regarding seasonal variations in neonatal mortality are temperature differentials between seasons, which vary across locations. In southern Europe, neonatal mortality was higher in the winter, whereas in eastern Europe and Russia, neonatal mortality was highest in the summer [29]. Other factors influencing the seasonality of neonatal mortality is the seasonality of human activities. A mother’s agricultural work during the harvest season might be associated with earlier weaning or lack of infant care [29]. There is also a documented seasonality of infectious diseases (peaks of respiratory diseases in winter and digestive diseases in summer). In their study of neonatal mortality in northern Italy from 1820 to 1900, Scalone and Samoggia [20] used daily temperature data as time-constant (temperature at birth) and time-varying (temperature after birth) variables. Their findings revealed that the effect of cold temperature at birth varied according to socioeconomic status, with the highest cold-related mortality risk found among the infants of landless rural laborer’s [20]. In the Netherlands, Ekamper et al. [19] showed that, from 1850 to 1954, the relationship between daily average temperature and age-specific mortality was strongest among infants (<1 year), in which both extremely high and low temperatures significantly increased the risk of infant mortality.

One important effect modifier of the association between temperature and neonatal mortality is adverse birth outcomes, such as low birth weight, low gestational age and malformations. For example, cold temperatures during the neonatal period primarily increase the risk of cold-related infectious diseases among preterm fragile infants [20]. Generally, preterm births and low birth weight are birth outcomes that are more common among boys [30,31,32], which increases the risk of neonatal mortality and cold-related causes of death such as hypothermia [33]. Slower lung maturation among male infants has been shown to be a major factor behind the male disadvantage in neonatal survival [34]. Recent research regarding the gender difference in weather vulnerability has shown a beneficial effect of increased temperature on survival among male infants [35].

### 1.2. Seasonality, Temperature and Neonatal Mortality in Preindustrial Sweden

Previous studies of infant mortality between Sami and non-Sami populations have found generally higher mortality rates among the Sami population compared to the non-Sami population, as well as significant differences in neonatal deaths between parishes in the Sápmi area [4,12]. In the nineteenth century, the Sami experienced the highest neonatal mortality rate during the winter (December–February) [7]. The non-Sami population showed no clear seasonal pattern in neonatal mortality, yet a higher mortality was found among infants born in January and November [7]. Furthermore, our previous research showed an association between extreme winter temperatures and neonatal mortality between 1800 and 1900, during which time neonatal mortality increased during the cold winter months (monthly mean temperatures below −15 °C) compared to the milder winter months, particularly among the Sami population [6]. We also found a decreased vulnerability in the Sami population during the second half of the century, suggesting improvements in overall living conditions and health outcomes [6].

Following previous research on gender differences in neonatal mortality, we hypothesized that weather vulnerability was higher among winter-born male infants compared to female infants. Further, we hypothesized that the weather vulnerability of winter-born infants was higher among the Sami population compared to the non-Sami population because of their semi-nomadic lifestyle.

## 2. Materials and Methods

### 2.1. Population Data

The source material for this study comprises digitized parish records provided by the Demographic Data Base at Umeå University [36]. The database contains linked individual data from parish registers and registers of birth, deaths, migration and so forth and includes every individual who was born or migrated to the parishes [37]. The present study covers the period from 1860 to 1899.

Neonatal deaths are defined as deaths that occur during the first 28 days of life (stillbirths excluded). In order to study the association between temperature and neonatal deaths, information was obtained about the birth and death date of every individual born in the two parishes between 1860 and 1899. Historical population data (such as this) often lack the birth and death data of every individual and, in such cases, any individuals with missing data have been excluded from our analyses. Compared to other age groups, there is a risk that neonatal deaths have been underreported [2]. However, previous studies of neonatal mortality in Sápmi between 1800 and 1900 found that around 92% of the population had registered birth and death dates [7]. The starting year used in this study (1860) corresponds to the year when the state authority, Statistics Sweden, was formed, which resulted in even more accurate reporting of demographic data [38].

Based on information about family name, parish, occupation and family relations, an ethnical indicator that distinguished between the Sami population and the non-Sami population has been developed [39]. This ethnical indictor has been used in a variety of studies regarding marriage, fertility and mortality patterns in Swedish Sápmi [4,6,7,8,13,23,39,40]. The available information about ethnicity does not allow a differentiation between nomadic and settled Sami. Even if there was an increase in Sami who became settled during the nineteenth century, the trend to settle was weaker in this northern part of Sápmi compared to the southern part [41]. During our study period 1860 and 1899, the majority of Sami still lived from a combination of reindeer herding, hunting, fishing and small-scale farming [41] and also those settled often took an active part in the reindeer herding [23,39].

Each infant was categorized based on its ethnicity, gender, season of birth and age. Two individuals of “unknown” gender were treated as missing values in the analyses. In the models, we controlled for the age of the neonates, categorized as—“first week,” “second week,” “third week and later.” Season of birth was categorized as winter (December–February), spring (March–May), summer (June–August) and autumn (September–November).

### 2.2. Temperature Data

**Figure 1:**
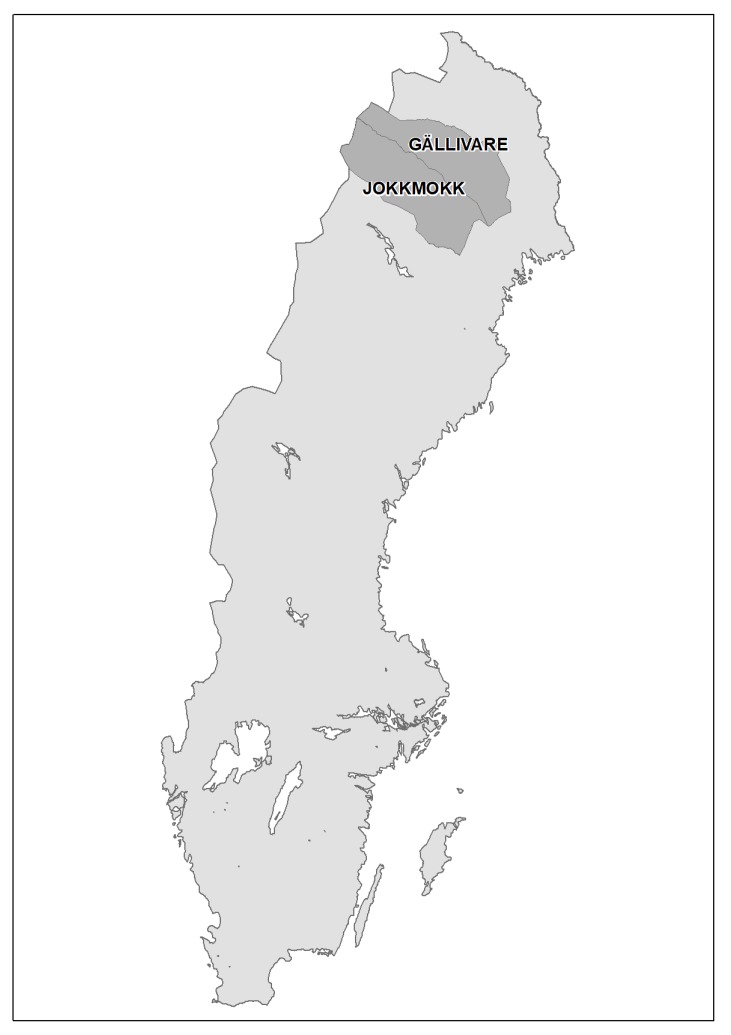
Map of Sweden including the study area of Jokkmokk and Gällivare. Reproduced with permission from the Swedish National Archives.

The regular recording of daily temperature started in Sweden between 1858 and 1860. Prior to this, records of daily temperature were collected on a less regular basis by a variety of actors [42]. In this study, we used weather data from Jokkmokk and Gällivare, provided by the Swedish Meteorological and Hydrological Institute (SMHI) [42]. These data contain daily temperature measurements taken in the morning, at noon and in the evening. Data that have been controlled for quality by SMHI were available for Jokkmokk (April 1879–December 1899) and for Gällivare (November 1888–December 1899) but contained some gaps. Non-controlled data were available for Jokkmokk starting in November 1860. Daily mean temperature was calculated based on the three daily measurements. If one of them was missing, all available measurements on the same day, the day before (lag) and the day after (lead) were used. Jokkmokk measurements replaced missing data in the Gällivare records. Geographically, Jokkmokk and Gällivare are adjacent to each other (see Figure 1) and we expected no major differences in daily temperatures between the two parishes. Mean daily temperatures for the study region combining Jokkmokk and Gällivare were calculated based on the best available measurement. All temperature values in this study are given in degrees Celsius (°C).

Since the aim of the study was to investigate the association between neonatal mortality and temperature at birth and after birth, for example, if lower temperatures on the day of birth or following birth significantly increased the risk of neonatal death, potential bias in the temperature data is not assumed to distort the analyses.

Table 1 shows summary statistics of minimum, maximum and mean temperatures during the study period from 1860 to 1899 in Jokkmokk and Gällivare, calculated by daily temperatures. Swedish Sápmi has a subarctic climate (long, cold winters and short, mild summers) and temperatures varied considerably between the lowest value of −40 °C (February) and the highest value of 26.2 °C (June). As seen in Table 1, values of less than 0 °C were found during virtually the entire year, except for July and August. The winter months, December through February, had the lowest minimum and mean temperatures, followed by November and March. In the following analyses of the association between temperature and neonatal mortality during the cold months, November through March were selected as cold winter months. In the analyses, we differentiated between temperature on the day of birth and daily temperature during the 28 days after birth (unless the infant died within this period).

### 2.3. Longitudinal Dataset

The population data and temperature data were merged into a longitudinal person-day dataset containing daily information of each infant born in the two parishes from 1860 to 1899. In the longitudinal dataset, each child was followed from the day of birth to the 28th day of life (unless the infant died within this period), complemented by temperature at birth (as a constant) and daily temperature. The longitudinal dataset includes the demographic characteristics of each infant, such as gender, age and ethnicity. We have two different study samples—a total group for seasonality analyses and a winter-born group for temperature analyses (see Table 2).

### 2.4. Data Analysis

All analyses were conducted following a time-event binomial regression model using a complementary log-log link function (hereafter called discrete-time regression), commonly used in survival analysis of binary events measured at discrete time intervals [43]. First, we created models of the association between season of birth and the risk of neonatal death according to ethnicity and gender. Second, we investigated the association between temperature (at birth and after) and the risk of neonatal death and its interaction with gender and ethnicity during the coldest months (November through March). The results are presented as hazard ratios (HR) with 95% confidence intervals (CI). All analyses were conducted using R statistical software (version 3. 4. 3, R Foundation for Statistical Computing, Vienna, Austria), survival, survminer and stargazer packages.

## 3. Results

### 3.1. Descriptives

In Jokkmokk and Gällivare parishes from 1860 to 1899, a total of 8024 live births were included in the analyses, of which 330 died during the neonatal period (4.1%) (Table 2). For analyses of neonatal mortality during the cold months (November through March), a total of 4007 live births were included, of which 159 were neonatal deaths (4.0%). In both study samples (whole group and winter-born), neonatal deaths were more common among males relative to females during the first week of life and were more common in Gällivare than in Jokkmokk.

### 3.2. Season and Neonatal Mortality

**Figure 2:**
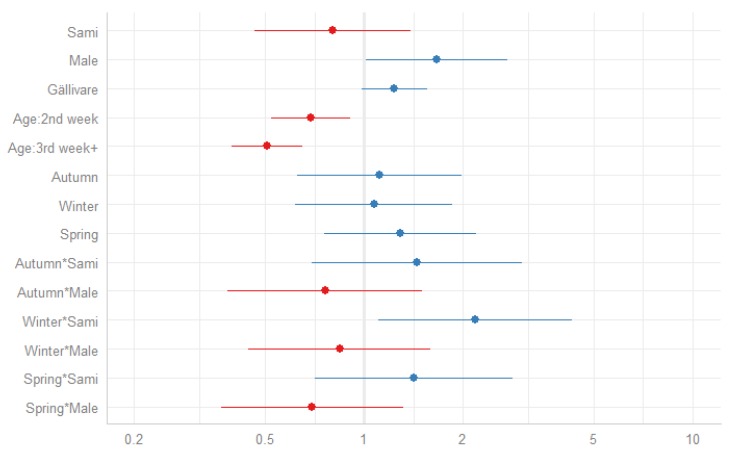
Hazard ratios of neonatal mortality with 95% confidence intervals (blue = increased hazard ratio, red = decreased hazard ratio), 1860–1899.

As a first step, the association between season and neonatal mortality was estimated in a stepwise manner. Estimates of all included parameters in the regression models are provided in the Appendix A (Table A1). In the base model (Model I), season of birth showed no major differences in neonatal mortality risk. However, infants born during the winter appeared to have a higher neonatal mortality risk compared to summer-born infants (HR 1.30, 0.95–1.77). Model V, including all of the parameters (excluding interactive terms), shows that male infants had a higher neonatal risk than females (HR 1.35, 1.08–1.68) and the neonatal mortality risk decreased in the following weeks during the first month of life (for age three to four weeks HR 0.51, 95% CI 0.40–0.65). In the last model (Model VI), including the interaction of season and ethnicity, being born in the winter had a stronger effect on the neonatal mortality risk among the Sami population (HR 2.18, 1.11–4.29) than non-Sami population. Compared to ethnicity, no seasonal effects were shown between the genders. The HR and their corresponding 95% confidence intervals for the last model (Model VI) are presented in Figure 2.

### 3.3. Temperature and Neonatal Mortality During an Extended Winter Season

**Figure 3:**
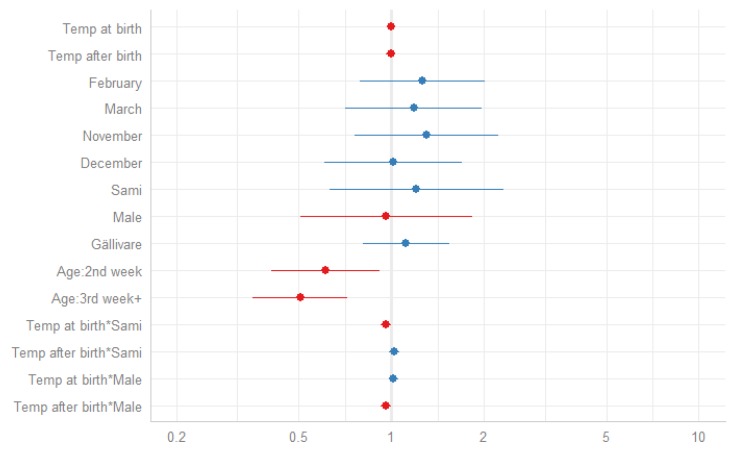
Hazard ratios of neonatal mortality with 95% confidence intervals (blue = increased hazard ratio, red = decreased hazard ratio), November–March, 1860–1899.

**Figure 4:**
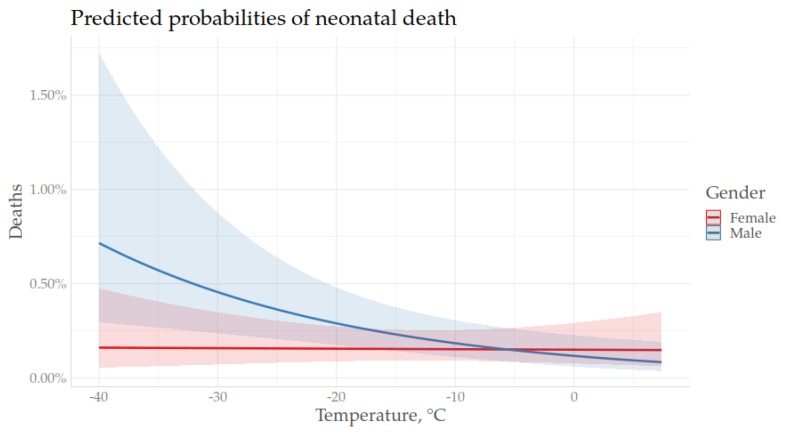
Predicted probabilities of neonatal mortality with 95% confidence intervals by temperature after birth, stratified by gender, November–March, 1860–1899.

**Figure 5:**
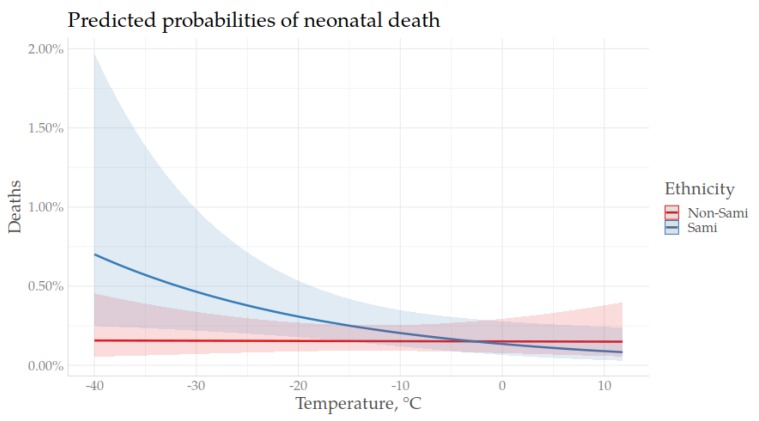
Predicted probabilities of neonatal mortality with 95% confidence intervals, by temperature at birth, stratified by ethnicity, November–March, 1860–1899.

As shown in previous models of the association between season of birth and neonatal mortality, being born during the winter was associated with a higher risk of neonatal mortality. In order to further investigate cold season mortality, the next analysis only included infants born during the coldest months, from November through March. As for the previous models, the association between temperature (at birth and following birth) and neonatal mortality was assessed in a stepwise manner, including the interactive terms of gender*temp and ethnicity*temp in the last model (Table A2, Appendix A). The base model (Model I), including only temperature at birth as a time-constant variable, showed no effect of temperature at birth on neonatal mortality risk. Including the daily mean temperature over the following 28 days (Model II) showed that neither of the two temperatures (time-constant or time-varying) had an effect on neonatal mortality. Model VII, including all of the parameters (excluding the interactive terms), showed that Sami infants had a higher neonatal mortality risk compared to winter born non-Sami infants (HR 1.46, 1.07–2.01) and the risk decreased during the neonatal period and was lowest at age three weeks and above (HR 0.51, 0.36–0.73). The last model (Model VIII) included all parameters and the interactive terms of temperature (as time-constant and time varying) and ethnicity, as well as temperature and gender. For the Sami, temperature at birth had a stronger effect than for non-Sami, where lower temperatures on the day of birth increased the risk. For male neonates, lower temperatures over the following 28 days had a stronger effect on neonatal mortality risk than on females, where lower temperatures after birth were associated with a higher risk of neonatal death. The relative risks with 95% confidence intervals of model VIII are presented in Figure 3. The predicted probabilities of neonatal death by temperature, stratified by gender and ethnicity, are presented in Figure 4 and Figure 5, respectively.

## 4. Discussion

During the first half of the nineteenth century, the Sami had higher neonatal mortality rates than the non-Sami population but from the mid nineteenth century onwards, the differences in infant and neonatal mortality between the two groups decreased [7,40]. Both populations had higher infant mortality rates than Sweden as a whole, implying that the colonization process had a negative effect on health for Sami and non-Sami [21].

In line with previous studies of seasonality and early infant mortality [24,44], this study showed that the overall neonatal mortality was higher in winter compared to summer. Comparing the Sami and the non-Sami population, the Sami winter born were more vulnerable, as confirmed by previous studies [7,13]. Unlike the study conducted by Scalone and Samoggia [20], this study found a mixed effect of temperature on neonatal mortality. Lower temperatures on the day of birth were related to a higher mortality risk among the Sami, whereas lower temperatures on the 28 days after birth were clearly associated with a higher mortality risk among male neonates.

Due to their semi-nomadic outdoor lifestyle, the Sami exposed their neonates to low temperatures and harsh weather conditions to a much higher extent than the non-Sami population (who lived in houses that were better protected from the cold). Contemporary observers have highlighted the combination of cold weather and migration associated with the nomadic life style as risk factors for the Sami neonates [45], where Sami women continued to migrate, sometimes directly after giving birth [12]. The Sami had strategies for protecting their infants from the cold. After being born, the infants were wrapped in a reindeer skin and were then placed in a *komse* (wooden cradle) that would protect them from hypothermia during the neonatal period [46]. In the nineteenth century, breast-feeding practices diverged in this northern region [47] and mixed-feeding practices were more common among non-Sami women than Sami women [12,48]. Sami women were used as a role model since they breastfed their children, sometimes for as long as 2–4 years, according to the local clergy [4,49]. It is likely that breast-feeding practices among Sami women and infant care after birth were protective factors, whereas the environmental circumstances and living conditions during birth (unheated houses, etc.) posed a risk factor, particularly during extremely cold periods.

The results showed that the risk of neonatal mortality was highest during the early neonatal period (first week of life) compared to the latter part. Controlling for the age of the neonates, the effect of temperature on the day of birth for the Sami appears to have had a debilitating effect on Sami neonate’s survival, even in the days following birth. As suggested by Scalone and Samoggia [20], low temperatures on the day of birth made it more difficult for neonates to maintain their body temperature which, in turn, risked weight loss on the first day after birth, followed by increased vulnerability to hypothermia.

An interesting result of this study was the greater mortality effect of temperature on the days after birth for males than females. Compared to season of birth, in which winter was associated with an overall higher mortality risk, this study found no gender difference in seasonality. It can be expected that the negative effect of low temperatures following birth among males is associated with a higher risk of being both preterm and underweight, as these two factors increase the risk of hypothermia [16].

Between the second half of the nineteenth century and the first decades of the twentieth century, the neonatal mortality rate in Sweden did not decrease to the same extent as the infant mortality rate [50,51]. The reason why the neonatal mortality rate lagged behind can be explained by a number of factors that affect the two rates in different ways. Improved living standards, better housing, nutrition and hygiene are factors that have contributed the most to the decrease in infant mortality, particularly towards the end of the first year of life, whereas causes of death associated with pregnancy and delivery account for a high proportion of neonatal mortality [50]. During the time frame of this study (1860–1899) and in this northern hinterland of Sweden, giving birth was still a private matter in your own home and was sometimes assisted by midwives. A potential area of research is whether the effect of season of birth and temperature decreased as higher proportions of births became institutionalized during the first half of the nineteenth century. Another potential area of research is the way in which the effect of temperature (both at birth and following birth) might differ between socio-economic strata at the onset of industrialization and urbanization. Previous studies have shown socio-economic differences in the rates of infant and perinatal mortality and the incidence of low birth weight [52], contributing to social inequalities in cold-related mortality. Unlike most contemporary research, our data lacked information on gestational age and birthweight. Since preterm birth, stillbirth and low birth weight are birth outcomes that are more prevalent in winter and summer [53], extreme temperature might also be an important determinant of poor birth outcomes.

This study has revealed the association between season and temperature and neonatal mortality in a subarctic region during the latter part of the nineteenth century. As hypothermia is still a serious problem in countries in which the neonatal mortality rate is high, our findings will provide important insights into factors that contribute to neonatal health and survival. The effect of temperature during the neonatal period among males in particular should be investigated in contemporary low-income societies. Our findings can also be communicated to high-income countries that already have low neonatal mortality rates, where the time spent by mothers and new-borns at hospital is decreasing, sometimes to a couple of hours after delivery or when the nearest hospital is far away, risking the mother giving birth outside hospital.

### Strengths and Limitations

Previous research of cold-related mortality has mainly used season of birth as a proxy for temperature or monthly mean temperatures from remote weather stations [6,7,8]. To our knowledge, this is the first study to examine the association between temperature and neonatal mortality in Sweden during the nineteenth century using local daily temperature data. In this study, we have focused on temperature effects during the cold months (November–March). A different approach would have been to examine the effect of cold and warm temperatures during different seasons, for example, the effect of cold temperature in summer compared to other seasons and warm temperature in winter compared to other seasons (summer).

## 5. Conclusions

The results showed that Sami neonatal mortality was higher during winter and that the Sami neonatal mortality risk decreased with higher temperatures on the day of birth. Male neonatal risk decreased with higher temperatures during the days following birth, while no effect of temperature was observed among female neonates. We conclude that weather vulnerability differed between genders and between the indigenous and non-indigenous populations.

## Figures and Tables

**Table 1:** Descriptive statistics of temperature in °C, 1860–1899.

Month	Min	Mean	Max	SD
January	−39.3	−14.8	4.5	8.6
February	−40.0	−13.3	4.7	8.4
March	−27.0	−7.8	6.7	6.2
April	−13.2	−0.5	10.4	4.1
May	−5.9	5.5	20.5	4.4
June	0.0	12.4	26.2	4.8
July	2.3	15.0	25.7	3.7
August	2.4	12.3	25.7	3.2
September	−3.7	6.6	15.7	3.6
October	−23.5	−1.3	12.7	5.7
November	−31.2	−8.9	7.4	7.5
December	−39.7	−13.5	5.3	8.6

**Table 2:** Descriptive statistics of population data, 1860–1899.

Total (%)	Neonatal Mortality, January–December	Neonatal Mortality in Winter-Born (November–March)
Total number of live births	8024	4007
Total number of neonatal deaths	330 (4.1%)	159 (4.0%)
Neonatal deaths by:		
Ethnicity		
Sami	121 (36.7%)	66 (41.5%)
Non-Sami	209 (63.3%)	93 (58.5%)
Gender		
Male	190 (57.6%)	91 (57.2%)
Female	140 (42.4%)	68 (42.8%)
Parish		
Jokkmokk	115 (34.8%)	58 (36.5%)
Gällivare	215 (65.2%)	101 (63.5%)
Age at death		
First week	124 (37.6%)	62 (39%)
Second week	84 (25.5%)	37 (23.3%)
Third week+	122 (37.0%)	60 (37.7%)
Season		
Winter (Jan–Feb)	98 (29.7%)	98 (61.6%)
Spring (March–May)	94 (28.5%)	33 (20.8%)
Summer (June–Aug)	68 (20.6%)	
Autumn (Sep–Nov)	70 (21.2%)	28 (17.6%)

## Appendix A

**Table A1:** Discrete time regression models of neonatal mortality by season, with interactions gender*seasons and ethnicity*seasons, expressed as hazard ratios, confidence intervals (CI 95%) and *p*-value, 1860–1899.

*Predictors*	Model I			Model II			Model III			Model IV			Model V			Model VI		
	*HR*	*CI*	*p*	*HR*	*CI*	*p*	*HR*	*CI*	*p*	*HR*	*CI*	*p*	*HR*	*CI*	*p*	*HR*	*CI*	*p*
Autumn	1.06	0.76–1.49	0.714	1.06	0.76–1.48	0.725	1.06	0.76–1.48	0.739	1.05	0.76–1.47	0.755	1.05	0.75–1.47	0.765	1.12	0.63–1.98	0.710
Spring	1.16	0.85–1.59	0.347	1.15	0.84–1.57	0.372	1.16	0.85–1.58	0.363	1.15	0.84–1.58	0.367	1.15	0.84–1.57	0.376	1.29	0.76–2.20	0.346
Winter	1.30	0.95–1.77	0.099	1.29	0.94–1.75	0.111	1.29	0.95–1.76	0.107	1.28	0.94–1.75	0.113	1.27	0.93–1.73	0.128	1.07	0.62–1.86	0.802
Sami				1.24	0.99–1.55	0.061	1.24	0.99–1.55	0.059	1.23	0.98–1.54	0.074	1.23	0.98–1.53	0.075	0.80	0.46–1.39	0.435
Male							1.36	1.09–1.69	0.006	1.35	1.09–1.68	0.007	1.35	1.08–1.68	0.007	1.67	1.02–2.73	0.042
Gällivare										1.24	0.99–1.56	0.064	1.24	0.99–1.55	0.064	1.24	0.99–1.55	0.064
Age: 2nd week													0.69	0.52–0.91	0.008	0.69	0.52–0.91	0.008
Age: 3rd week+													0.51	0.40–0.65	<0.001	0.51	0.40–0.65	<0.001
Autumn*Sami																1.45	0.69–3.03	0.326
Spring*Sami																1.42	0.71–2.83	0.324
Winter*Sami																2.18	1.11–4.29	0.024
Autumn*Male																0.76	0.38–1.51	0.434
Spring*Male																0.70	0.37–1.32	0.265
Winter*Male																0.84	0.45–1.59	0.597
Observations	218897			218897			218897			218897			218897			218897		
R^2^ Nagelkerke	0.001			0.001			0.003			0.004			0.009			0.011		

**Table A2:** Discrete time regression models of neonatal mortality by temperature, with interactions sex*temperature and ethnicity*temperature, expressed as hazard ratios, confidence intervals (CI 95%) and *p*, cold seasons (November–March), 1860–1899.

	Model I			Model II			Model III			Model IV			Model V			Model VI			Model VII			Model VIII		
*Predictors*	*HR*	*CI*	*p*	*HR*	*CI*	*p*	*HR*	*CI*	*p*	*HR*	*CI*	*p*	*HR*	*CI*	*p*	*HR*	*CI*	*p*	*HR*	*CI*	*p*	*HR*	*CI*	*p*
Temp at birth	0.99	0.97–1.01	0.163	0.99	0.97–1.01	0.373	0.99	0.97–1.01	0.309	0.99	0.97–1.01	0.321	0.99	0.97–1.01	0.341	0.99	0.97–1.01	0.347	0.99	0.97–1.01	0.408	1.00	0.97–1.03	0.957
Temp after birth				0.99	0.97–1.00	0.132	0.98	0.96–1.00	0.107	0.98	0.96–1.00	0.107	0.98	0.96–1.00	0.108	0.98	0.96–1.00	0.108	0.98	0.96–1.00	0.120	1.00	0.96–1.03	0.922
February							1.24	0.78–1.98	0.365	1.25	0.78–2.00	0.347	1.26	0.79–2.00	0.340	1.25	0.79–2.00	0.344	1.26	0.79–2.01	0.329	1.26	0.79–2.02	0.325
March							1.15	0.69–1.91	0.587	1.16	0.70–1.93	0.561	1.16	0.70–1.92	0.568	1.16	0.70–1.92	0.569	1.17	0.70–1.94	0.548	1.18	0.71–1.97	0.520
November							1.32	0.77–2.26	0.316	1.33	0.77–2.27	0.304	1.32	0.77–2.26	0.309	1.32	0.77–2.26	0.312	1.31	0.77–2.24	0.318	1.30	0.76–2.22	0.338
December							1.03	0.62–1.71	0.917	1.03	0.61–1.71	0.922	1.03	0.62–1.71	0.918	1.03	0.61–1.71	0.922	1.02	0.61–1.70	0.946	1.01	0.61–1.69	0.959
Sami										1.47	1.07–2.02	0.016	1.48	1.08–2.03	0.015	1.47	1.07–2.02	0.017	1.46	1.07–2.01	0.018	1.21	0.63–2.32	0.569
Male													1.34	0.98–1.83	0.070	1.33	0.97–1.83	0.073	1.33	0.97–1.83	0.072	0.96	0.51–1.83	0.910
Gällivare																1.11	0.80–1.53	0.531	1.11	0.80–1.53	0.530	1.12	0.81–1.55	0.498
Age: 2nd week																			0.61	0.41–0.92	0.019	0.61	0.41–0.92	0.018
Age: 3rd week+																			0.51	0.36–0.73	<0.001	0.51	0.35–0.72	<0.001
Temp at birth*Sami																						0.96	0.93–1.00	0.036
Temp after birth*Sami																						1.03	0.99–1.07	0.193
Temp at birth*Male																						1.02	0.98–1.06	0.350
Temp after birth*Male																						0.96	0.92–1.00	0.028
Observations	93368			93368			93368			93368			93368			93368			93368			93368		
R^2^ Nagelkerke	0.001			0.002			0.002			0.005			0.006			0.007			0.012			0.017		

## References

[1] Sundin J., Sundin J., Statens F. (2005). Svenska Folkets Hälsa I Historiskt Perspektiv.

[2] Bengtsson T. (1999). The vulnerable child. Economic insecurity and child mortality in pre-industrial Sweden: A case study of Vastanfors, 1757–1850. Eur. J. Popul..

[3] Edvinsson S. (1992). Den Osunda Staden Sociala Skillnader I Dödlighet I 1800-Talets Sundsvall.

[4] Sköld P., Axelsson P., Karlsson L., Smith L. (2011). Infant mortality of Sami and settlers in Northern Sweden: The era of colonization 1750–1900. Glob. Health Action.

[5] Edvinsson S., Brändström A., Rogers J., Lars-Göran T., Peter S. (2001). Regional variations in infant mortality in Sweden during the first half of the 19th century. Nordic Demography in History and Present-Day Society.

[6] Karlsson L., Lundevaller E., Schumann B. (2019). The association between cold extremes and neonatal mortality in Swedish Sápmi from 1800 to 1895. Glob. Health Action.

[7] Karlsson L., Lundevaller E.H., Schumann B. (2019). Season of birth, stillbirths, and neonatal mortality in Sweden: The Sami and non-Sami population, 1800–1899. Int. J. Circumpolar Health.

[8] Schumann B., Haggstrom Lundevaller E., Karlsson L. (2019). Weather extremes and perinatal mortality - Seasonal and ethnic differences in northern Sweden, 1800–1895. PLoS ONE.

[9] Coory M. (2003). Can a mortality excess in remote areas of Australia be explained by Indigenous status? A case study using neonatal mortality in Queensland. Aust. N. Z. J. Public Health.

[10] Luo Z.-C., Wilkins R., Heaman M., Smylie J., Martens P.J., McHugh N.G.L., Labranche E., Simonet F., Wassimi S., Minich K. (2012). Birth outcomes and infant mortality among First Nations Inuit, and non-Indigenous women by northern versus southern residence, Quebec. J. Epidemiol. Commun. H.

[11] Smylie J., Crengle S., Freemantle J., Taualii M. (2010). Indigenous Birth Outcomes in Australia, Canada, New Zealand and the United States—An Overview. Open Womens Health J..

[12] Brändström A., Åkerman S., Lundholm K. (1990). Från förebild till motbild. Spädbarnsvård och spädbarnsdödlighet i Jokkmokk. Älvdal I Norr Människor Och Resurser I Luledalen 1300–1800.

[13] Karlsson L. (2017). Indigenous Infant Mortality by Age and Season of Birth, 1800–1899: Did Season of Birth Affect Children’s Chances for Survival?. Int. J. Environ. Res. Public Health.

[14] Naeye R.L., Burt L.S., Wright D.L., Blanc W.A., Tatter D. (1971). Neonatal mortality, the male disadvantage. Pediatrics.

[15] Yerushalmy J. (1938). Neonatal mortality by order of birth and age of parents. Am. J. Hyg..

[16] Zhao D., Zou L., Lei X., Zhang Y. (2017). Gender Differences in Infant Mortality and Neonatal Morbidity in Mixed-Gender Twins. Sci. Rep..

[17] Yaya S., Diarra S., Mabeu M.C., Pongou R. (2018). The sex gap in neonatal mortality and the AIDS epidemic in sub-Saharan Africa. BMJ Glob. Health.

[18] Mizuno R. (2000). The male/female ratio of fetal deaths and births in Japan. Lancet.

[19] Ekamper P., van Poppel F. (2019). Infant mortality in mid-19th century Amsterdam: Religion, social class, and space. Popul. Space Place.

[20] Scalone F., Samoggia A. (2018). Neonatal mortality, cold weather, and socioeconomic status in two northern Italian rural parishes, 1820–1900. Demogr. Res..

[21] Sköld P., Axelsson P. (2008). The northern population development; Colonization and mortality in Swedish Sapmi, 1776–1895. Int. J. Circumpolar Health.

[22] Axelsson P., Sköld P. (2006). Indigenous Populations and Vulnerability. Characterizing Vulnerability in a Sami Context. Ann. Démographie Hist..

[23] Nordin G., Sköld P. (2012). True or false? Nineteenth-century Sápmi fertility in qualitative vs. demographic sources. Hist. Fam..

[24] Breschi M., Derosas R., Manfredini M., Bengtsson T., Campbell C., Lee J. (2004). Mortality and Environment in Three Emilian, Tuscan, and Venetian Communities, 1800–1883. Life under Pressure: Morality and Living Standards in Europe and Asia, 1700–1900.

[25] Breschi M., Livi-Bacci M., Desjardins B. (1997). Month of Birth as a Factor in Children’s Survival. Infant and Child Mortality in the Past Oxford.

[26] Dalla-Zuanna G., Rosina A. (2011). An Analysis of Extremely High Nineteenth-Century Winter Neonatal Mortality in a Local Context of Northeastern Italy. Eur. J. Popul..

[27] Derosas R. (2009). The joint effect of maternal malnutrition and cold weather on neonatal mortality in nineteenth-century Venice: An assessment of the hypothermia hypothesis. Pop. Stud. J. Demog..

[28] Reher D.S., Gimeno A.S. (2006). Marked from the outset: Season of birth and health during early life in Spain during the demographic transition. Contin. Chang..

[29] Breschi M., Pozzi L. (2004). The Determinants of Infant And Child Mortality in Past European Populations.

[30] Astolfi P., Zonta L.A. (1999). Risks of preterm delivery and association with maternal age, birth order, and fetal gender. Hum. Reprod..

[31] Ingemarsson I. (2003). Gender aspects of preterm birth. BJOG.

[32] Mohamed M.A., Aly H. (2018). Would fetal sex affect the odds for premature delivery?. Pediatrics.

[33] Miller S.S., Lee H.C., Gould J.B. (2011). Hypothermia in very low birth weight infants: Distribution, risk factors and outcomes. J. Perinatol..

[34] Khoury M.J., Marks J.S., McCarthy B.J., Zaro S.M. (1985). Factors affecting the sex differential in neonatal mortality: The role of respiratory distress syndrome. Am. J. Obs. Gynecol..

[35] Babalola O., Razzaque A., Bishai D. (2018). Temperature extremes and infant mortality in Bangladesh: Hotter months, lower mortality. PLoS ONE.

[36] DDB (2019). Demographic Data Base.

[37] Westberg A., Engberg E., Edvinsson S. (2016). A Unique Source for Innovative Longitudinal Research: The POPLINK Database. Hist. Life Course Stud..

[38] Woods R. (2009). Death before Birth: Fetal Health and Mortality in Historical Perspective.

[39] Nordin G. (2009). Äktenskap i Sápmi: Giftermålsmönster Och Etnisk Komplexitet I Kolonisationens Tidevarv, 1722–1895.

[40] Karlsson L. (2013). Indigenous life expectancy in Sweden 1850–1899: Towards a long and healthy life?. Demogr. Res..

[41] Brännlund I. (2018). Diverse Sami Livelihoods: A Comparative Study of Livelihoods in Mountain-Reindeer Husbandry Communities in Swedish Sápmi 1860–1920. J. North. Stud..

[42] (2019). Swedish Meteorological and Hydrological Institute. www.smhi.se.

[43] Abbott R.D. (1985). Logistic regression in survival analysis. Am. J. Epidemiol..

[44] Oris M., Derosas R., Breschi M. (2004). Infant and Child Mortality Life under Pressure: Mortality and Living Standards in Europe and Asia, 1700–1900.

[45] Düben G.V. (1873). Om Lappland Och Lapparne, Företrädesvis De Svenske: Ethnografiska Studier.

[46] Serning I. (1950). Lappbarnen, Deras Vård och Uppfostran i Spädbarnsåldern och Lekåldern. 1949 s. 55–109.

[47] Thorvaldsen G. (2008). Was there a European breastfeeding pattern?. Hist. Fam..

[48] Brändström A. (1993). Infant Mortality in Sweden 1750–1950: Past and Present Research into Its Decline.

[49] Ellmin J. (1851). Annual Report. District Physician of Jämtland.

[50] Kohler L. (1991). Infant-Mortality—The Swedish Experience. Annu. Rev. Publ. Health.

[51] Wallgren A. (1942). The neonatal mortality in Sweden from a pedlatric point of view. Acta Paediatr..

[52] Zetterström R., Eriksson M. (1987). Hälsa och Social Klass: Spädbarnsdödlighet och Graviditetsutfall. Soc. Med. Tidskr..

[53] Strand L.B., Barnett A.G., Tong S. (2011). The influence of season and ambient temperature on birth outcomes: A review of the epidemiological literature. Environ. Res..

